# A Systematic Review of the Association Between Vegan Diets and Risk of Cardiovascular Disease

**DOI:** 10.1093/jn/nxab037

**Published:** 2021-04-08

**Authors:** Jeenan Kaiser, Kim R van Daalen, Arjun Thayyil, Mafalda Tasso de Almeida Ribeiro Reis Cocco, Daniela Caputo, Clare Oliver-Williams

**Affiliations:** Department of Public Health and Primary Care, University of Cambridge, Cambridge, United Kingdom; Faculty of Medicine and Dentistry, University of Alberta, Edmonton, Canada; Cardiovascular Epidemiology Unit, Department of Public Health and Primary Care, Strangeways Research Laboratory, Cambridge, United Kingdom; School of Clinical Medicine, University of Cambridge, Cambridge, United Kingdom; School of Clinical Medicine, University of Cambridge, Cambridge, United Kingdom; National Institute for Health Research BioResource, Addenbrooke's Hospital, Cambridge, United Kingdom; Cardiovascular Epidemiology Unit, Department of Public Health and Primary Care, Strangeways Research Laboratory, Cambridge, United Kingdom; Homerton College, University of Cambridge, Cambridge, United Kingdom; Department of Health Sciences, University of Leicester, Leicester, United Kingdom

**Keywords:** vegan, plant-based, cardiovascular disease, coronary heart disease, coronary artery disease, stroke, ischemic attack, carotid artery intima-media thickness, dietary interventions, public health

## Abstract

**Background:**

Plant-based diets are gaining attention globally due to their environmental benefits and perceived health-protective role. A vegan diet may have cardiovascular benefits; however, evidence remains conflicting and insufficiently assessed.

**Objectives:**

We evaluated the utility of the vegan diet in cardiovascular disease (CVD) prevention.

**Methods:**

We conducted a systematic review of studies evaluating the association between vegan diets and cardiovascular outcomes. We searched 5 databases (Ovid MEDLINE, EMBASE, Web of Science, Scopus, and OpenGrey) through 31 October 2020. Four investigators independently screened the full texts for inclusion, assessed quality, and extracted data from published reports.

**Results:**

Out of the 5729 identified records, 7 were included, comprising over 73,000 participants, of whom at least 7661 were vegans. Three studies, with at least 73,426 individuals (including at least 7380 vegans), examined risks of primary cardiovascular events (total CVD, coronary heart disease, acute myocardial infarction, total stroke, hemorrhagic stroke, and ischemic stroke) in individuals who followed a vegan diet compared to those who did not. None of the studies reported a significantly increased or decreased risk of any cardiovascular outcome. One study suggested that vegans were at greater risk of ischemic stroke compared to individuals who consumed animal products (HR, 1.54; 95% CI, 0.95–2.48). Yet in another study, vegans showed lower common carotid artery intima-media thickness (0.56 ± 0.1 mm vs. 0.74 ± 0.1 mm in controls; *P* < 0.001), and in 3 studies of recurrent CVD events, vegans had 0–52% lower rates. Furthermore, endothelial function did not differ between vegans and nonvegans. Using the Grading of Recommendations Assessment, Development and Evaluation approach, evidence was deemed to be of low to very low strength/quality.

**Conclusions:**

Among the Western populations studied, evidence weakly demonstrates associations between vegan diets and risk of CVDs, with the direction of associations varying with the specific CVD outcome tested. However, more high-quality research on this topic is needed. This study was registered at PROSPERO as CRD42019146835.

## Introduction

Plant-based diets have increased in popularity due to concerns for the environment and animal welfare and due to perceived health benefits (1–3). The Food and Agriculture Organization (FAO) indicates that adopting sustainable diets is essential to addressing the degradation of environmental resources and climate change (4). As global livestock is considered to be responsible for 18% of anthropogenic greenhouse gas (GHG) emissions, this includes diets that reduce animal product consumption and increase plant consumption (5). Such plant-based diets are associated with reduced diet-related GHG emissions, land use associated with food production, and cumulative energy demand indicators, regardless of the level of plant-based food consumption (2, 3, 6). Furthermore, accumulating evidence on the potential health benefits of plant-based diets has made them of interest to researchers, health-care professionals, and public health practitioners, and may strengthen the case for the continued push towards sustainable diets to promote not only environmental sustainability but also population health (1, 7, 8). Moreover, during the coronavirus disease pandemic, plant-based diets have been proposed as a potential means to prevent and mitigate future transmission of viruses between different species and humans (9). However, the merits of plant-based diets relative to other diets in managing and preventing cardiovascular disease (CVD) has been the subject of intense debate (10).

Although there is significant heterogeneity in the types and specific definitions of plant-based diets, veganism, specifically, is defined as the complete exclusion of animal products, including meat, fish, poultry, seafood, dairy, and eggs (7, 11). The vegan diet has been found to be significantly associated with beneficial changes in cardiometabolic CVD risk factors, such as lower BMIs, serum total cholesterol levels, serum glucose levels, inflammation, and blood pressure, compared to omnivorous diets, which are typically lower in whole grains, fruits, nuts, and vegetables (7, 12, 13). Such positive cardiovascular health effects may result from lower intakes of dietary cholesterol, saturated fat, and red and processed meat, as well as higher intakes of fiber, plant protein, and phytonutrients (14, 15). These observations suggest that the vegan diet may have therapeutic potential in preventing or treating CVD.

Contrastingly, levels of nutrients such as EPA, DHA, selenium, zinc, iodine, and vitamin B_12_ are significantly lower in vegans compared to nonvegans, which may similarly have adverse cardiovascular health effects (16, 17). Nevertheless, data on vegan diets are limited. Very few studies have evaluated vegan diets separately from other no-meat diets, such as ovo-lacto-vegetarianism, making it difficult to explore the specific effects of the vegan diet on cardiovascular health.

To date, no studies have compiled evidence for the role of the vegan diet alone on the risk of primary, intermediate, and recurrent CVD. A previous review conducted in 2015 evaluated only primary CVD, and therefore did not give a complete account of the effect of vegan diets on CVD (7). To address this knowledge gap, we performed a systematic review of studies assessing the association between vegan diets and risks of primary, intermediate, and recurrent CVD.

## Methods

### Study design

We conducted a systematic review of epidemiological studies exploring the relationships between vegan diets and primary, intermediate, and recurrent CVD risks, in accordance with the Preferred Reporting Items for Systematic Reviews and Meta-Analyses statement (17). This review was registered with PROSPERO as CRD42019146835.

### Search strategy and selection criteria

We systematically searched 5 databases, including a gray literature database (Ovid MEDLINE, EMBASE, Web of Science, Scopus and OpenGrey), through to 31 October 2020 using a controlled vocabulary. Search terms included those related to diet (e.g., veganism, plant-based), cardiovascular health (e.g., stroke, carotid-intima media thickness), and the relevant population (e.g., humans). We cross-referenced bibliographies of relevant publications identified in our search to capture any additional studies that fit our inclusion criteria (**Supplemental Table 1**).

The exposure of interest was adherence to a vegan diet, defined as no consumption of animal products or byproducts. By this definition, the vegetarian diet did not qualify as a vegan diet. Studies that met the inclusion criteria were written in any language, assessed humans, and examined associations between adherence to vegan diets and risks of CVD outcomes compared to a control group that followed a nonvegan diet. For studies that compared a vegan diet with several predefined diets (e.g., vegetarian diets and omnivorous diets), we considered the least restrictive diet as the comparison group (e.g., omnivorous diets).

Based on our exclusion criteria, nonhuman studies (e.g., animal or in vitro studies); studies that did not compare individuals who followed a vegan diet to individuals who followed a nonvegan diet; studies that did not evaluate a cardiovascular outcome; and studies that did not include any original analyses (e.g., reviews) were excluded. After removing duplicate records, the titles and abstracts of identified studies were screened according to the inclusion and exclusion criteria by 6 researchers (JK, KRvD, AT, MTdARRC, DC, and CO-W) using the software Abstrackr (18). All full texts for studies that satisfied all selection criteria were retrieved and screened (JK, KRvD, AT, and MTdARRC). Any divergences between authors on study eligibility were discussed and adjudicated by CO-W.

Data from included studies were independently extracted by 4 authors (JK, KRvD, AT, and MTdARRC). Discrepancies between authors were considered by CO-W and discussed among JK, KRvD, and CO-W until consensus was reached. The following information was extracted from each study: study design, study population, participant demographics, baseline characteristics, method of recruitment, details of vegan and control diets (e.g., definition and duration), total number of participants, numbers of vegan and nonvegan participants, reported outcomes, outcome ascertainment, number of events, and association measures with their 95% CIs. Where raw data were provided but no association measure given, the association measure and 95% CI were calculated. An open field to record additional relevant information was also included.

### Quality assessment

Four authors (JK, KRvD, AT, and MTdARRC) independently assessed the quality of studies using the Cochrane Collaboration Tool for randomized controlled trials (RCTs) (19) and the Newcastle-Ottawa Scale for cohort studies and cross-sectional studies (20). The final score was converted to Agency for Healthcare Research and Quality (AHRQ) standards. Discrepancies between authors were adjudicated by CO-W.

### Risk of bias across studies

To assess the risk of bias across individual studies, 3 authors (CO-W, JK, and KRvD) applied the Grading of Recommendations Assessment, Development and Evaluation (GRADE) approach (21). As individual studies were assessed using the Cochrane Collaboration's tool or the Newcastle-Ottawa Scale, the GRADE approach was only applied where 2 or more included studies evaluated the same outcome. Due to heterogeneity in the directions of the associations with different outcomes, we did not deem it appropriate to apply the GRADE approach to all of the studies as a single collective. Evidence from RCTs starts at high quality. In contrast, evidence from observational studies starts at low quality, due to residual confounding, among other issues. We downgraded the evidence by 1 level for each serious study limitation that was identified. Study limitations were risk of bias, indirectness of evidence, serious inconsistency, imprecision of effect estimates, or potential publication bias. Two or more studies were identified for the following outcomes and were therefore included in the GRADE assessment: primary coronary heart disease (CHD) (22, 23, 24), primary total stroke (22, 23) and recurrent CHD (25, 26).

### Outcomes

To provide a comprehensive assessment of CVD risks, our outcomes of interest included primary, intermediate, and recurrent CVD end points (**Supplemental Table 2**).

### Statistical analysis

A meta-analysis was deemed inappropriate due to high heterogeneity between studies.

## Results

### Included studies and baseline characteristics

**FIGURE 1:**
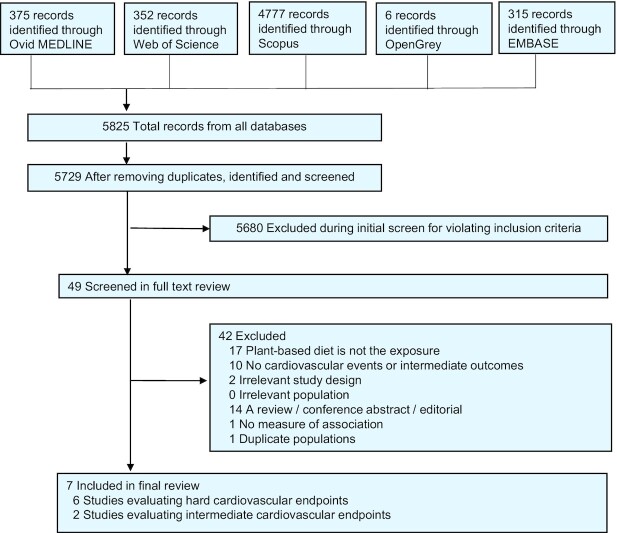
Study selection process. Hard cardiovascular end points included total cardiovascular disease, coronary heart disease, acute myocardial infarction, total stroke, hemorrhagic stroke, and ischemic stroke. Intermediate cardiovascular end points included carotid artery intima-media thickness and endothelial function.

The flow diagram in **Figure 1** depicts the search results from all databases. Characteristics of the 7 studies included in our systematic review are summarized in the **Tables 1** and **2**. Due to unspecified partially overlapping populations between 2 studies (22, 27) it was not possible to estimate the exact number of individuals included in the review, but there were at least 73,852 participants, of whom at least 7661 were vegan (12, 22–26, 28). Studies were conducted in the United States, (*n* = 4) (12, 24, 25, 28), the United Kingdom (*n* = 1) (23), and New Zealand (*n* = 1) (26), and 1 study combined data from the United States, Germany, and the United Kingdom (22). There were 4 prospective cohort studies (12, 22–24), 1 cross-sectional study (28), and 2 RCTs (25, 26). Cardiovascular events were ascertained through health service records (22, 23), the US National Death Index (22, 24), church records (22), data gathered through telephone or from personal contacts (12, 22, 25), and/or the registrar's office (22). Dietary assessments were conducted via FFQs (22–24), 24-hour dietary recalls (25), food diaries (25, 26, 28), and self-reported elimination of animal products (12).

**TABLE 1:** Included epidemiological studies examining the effect of a vegan diet on the risk of cardiovascular events^1^

Reference	Country	Population	Follow-up	Diet assessment	Vegan diet	Duration of time as vegan before study start	Control diet definition	Outcome assessment	Outcomes (types)	Outcomes (*n*)	Main findings^2^	Adjustment or matching
Prospective cohort studies												
Orlich et al., 2013 (24)	USA	40,907 participants from AHS-2 study*Mean age (y):* Vegan (*n* = 5,548): 57.9 ± 13.6Non-vegetarian (*n* = 35,359): 55.9 ± 13.1*Women:* 66%	5.8 y	Self-administered quantitative FFQ	Definition: No eggs, dairy, fish, all other meats <1 a monthMean duration: 21 y	Mean: 21 years	Non-fish meats >1 ×/mo and all meats combined >1 ×/wk	Mortality records from USA National Death Index	CVD deathsCHD deaths	987372	HR, 0.91; 95% CI, 0.71–1.16HR, 0.90; 95% CI, 0.60–1.33	Adjusted for age, sex, region, race, income, education, marital status, smoking, exercise, alcohol intake, sleep, menopausal status (females), HRT (if postmenopausal)
Tong et al., 2019 (23)	UK	26,260 participants from the EPIC-Oxford studyVegan (*n* = 1832)Meat-eater (*n* = 24,428)*Age range (y):* 35–59*Women:* 75%	Max: 18.1 y	Self-administered quantitative FFQ	Definition: No meat, fish, dairy, eggs in 2010 and at baselineMean duration: not reported	NG	Meat, regardless of whether they consumed fish, dairy, eggs	Hospital records	Acute MICHDStrokeHaemorrhagic strokeIschemic stroke	78828201072300519	HR, 0.77; 95% CI, 0.46–1.27HR, 0.82; 95% CI, 0.64–1.05HR, 1.35; 95% CI, 0.95–1.92HR, 1.09; 95% CI, 0.53–2.26HR, 1.54; 95% CI, 0.95–2.48	Adjusted for age, sex, region, recruitment method and year, education, Townsend deprivation index, smoking, alcohol intake, exercise, supplement use, HRT (females), oral contraceptive use
Key et al., 1999 (22)	USA, UK, Germany	32,519 participants from AM, HFS, AH, Heidelberg and Oxford vegetarian studies	11.7 y	Self-administered quantitative FFQ	Definition: No animal products	NG	Regular meat eaters consumed meat ≥1 ×/wk	Medical records linked to mortality records	CHD deaths	1743	HR, 0.74; 95% CI, 0.46–1.21	Adjusted for age, sex, smoking, study
		Vegan (*n* = 753)Meat-eater (*n* = 31,766)*Age range (y):* 16–89*Women:* not given			Mean duration: not reported				Cerebrovascular disease deaths (stroke)	617	HR. 0.70; 95% CI. 0.25–1.98	
Esselstyn et al., 2014 (12)	USA	198 self-selected patients counselled in plant-based nutrition at the CCP	44.2 mo	Self-reported elimination of dairy, fish, meat and added oils	Definition: No dairy, fish, meat, added oils; avoided avocado, nuts, excess salt, caffeine, fructose	0 days	Not reported	Self-report or next of kin	Improved angina, CAD progression, CVD deaths	144, 15, 2	1 major cardiac event and 17 interventions occurred in the adherent participants (10%) and 13 (62%) in nonadherent participants	Unadjusted
		Vegan (*n* = 177)			Mean duration: not reported							
		Non-vegan (*n* = 21)										
		*Mean age (y):* 62.9 ± 10.0										
		*Women:* 88%										
Cross-sectional studies												
Fontana et al., 2007 (28)	USA	3 groups of subjects^3^ (21 per group) recruited via the SLV society, RFOM, local advertising and endurance runners	N/A	7-d food record	Definition: No meat, dairy, eggs, cooked and processed foods	Range: 2–10 years	Typical Western diets	Ultrasound with transducer	Carotid artery IMT (mm)	N/A	IMT: 0.6 ± 0.1 versus 0.7 ± 0.1 in the low-calorie, low-protein, vegan diet group versus Western diet group, respectively (*P* < 0.001)	Adjusted for age, gender, BMI (endurance runners), height (healthy, sedentary, nonobese group)
		Vegan (*n* = 21)			Mean duration: 4.4 ± 2.8 y							
		Western diet (*n* = 42)										
		*Age range (y):* 5–35										
		*Women:* 38%										
Randomized controlled trials												
Shah et al., 2018 (25)	USA	100 (1:1) randomized participants from the EVADE CAD trial with a	8 wk	24-h dietary recall and 4-d food record	Definition: No meat, poultry, eggs, dairy, seafood Mean duration: 8 wk	0 days	AHA-recommended diet group encouraged to consume ≤5 oz of animal protein/day,	Self-report	MI, repeat coronary revascularization,	0	No events	N/A
		history of angiographically defined CAD					only low-fat/fat-free dairy if dairy was					
		*Median age (y):*Vegan (*n* = 50): 63.0 (57.0–68.0)					consumed, fish ≥2×/week	Determined by clinical neurologist consultant	Cerebrovascular disease	2	0 versus 2 events	N/A
		AHA diet (*n* = 50): 59.5 (53.0–67.0) *Women:* 15%						Measured using the EndoPat Device	Endothelial function	N/A	No significant change in endothelial function over time (vegans: baseline 1.96; 1.62, 2.70 at 8 weeks 1.88; 1.61, 2.61; *P* = 0.86; AHA diet: baseline 2.12; 1.85, 2.48 at 8 weeks 1.84; 1.68, 2.13; *P* = 0.12). No significant change in status of endothelial function (abnormal (<1.67) versus normal) was noted in 75% of vegan and 84% of AHA diet participants (*P* = 0.41); a change from normal to abnormal was found in 10% of vegan and 14% of AHA diet participants (*P* = 0.73) and a change from abnormal to normal in 15% of vegan and 3% of AHA diet participants (*P* = 0.11)	N/A
Wright et al., 2017 (26)	New Zealand	65 participants of the randomized BROAD randomised control trial Vegan (*n* = 33) Regular diet (*n* = 32) *Age range (y):* 35–70 *Women:* 60%	6–12 mo	3-d food recall	Definition: Low-fat plant-based diet including whole grains, legumes, vegetables, fruits. Mean duration: 1 y	0 days	Regular diet	Notification on transfer to higher cardiac-related care	CVD events	0	No cardiovascular events were recorded; the program led to significant improvements in CRP, BMI, cholesterol, and other risk factors	N/A

1 AH study, Adventist Health study; AHS-2, Adventist Health 2 Study; AM study, Adventist Mortality study; CAD, coronary artery disease; CCP, Cleveland Clinic Program; CHD, coronary heart disease; CVD, cardiovascular disease; EPIC-Oxford, European Prospective Investigation into Cancer and Nutrition; EVADE CAD, Effects of a Vegan versus American Heart Association–Recommend Diet in Coronary Artery Disease; HFS study, Health Food Shoppers study; HRT, hormone replacement therapy; IMT, intima-media thickness; MI, myocardial infarction; N/A, not applicable; NG, not given; RFOM, Raw Food Online Magazine; SLV Society, St. Louis Vegetarian Society.

2 Outcome comparing the adherence to a vegan dietary pattern to a nonvegan dietary pattern. The least restrictive diet was chosen as a comparison group (e.g., omnivorous diet).

3 The 3 groups consisted of a low-calorie low-protein vegan diet group, an endurance runner group and a Western diet group.

**TABLE 2:** Inclusion criteria, diet, and outcome ascertainment about the included studies^1^

Reference	Inclusion/exclusion criteria	Definition of vegan diet by study	Diet ascertainment	Details of diets of those not adhering to vegan diet	Definition of outcome	Method of outcome ascertainment
Prospective cohort studies						
Orlich et al., 2013 (24)	Exclusions: missing data for questionnaire return date, birth date, sex, or race (*n* = 1702); age younger than 25 years (*n* = 434); estimated energy intake (not including write-in items) <500 kcal/d or >4500 kcal/d; improbable response patterns (e.g., identical responses to all questions on a page) or >69 missing values in dietary data (*n* = 4961); non-US residents (*n* = 4108); or history of a specific prior cancer diagnosis (except nonmelanoma skin cancers) or of CVD (*n* = 11,956)	Vegans consumed eggs/dairy, fish, and all other meats less than 1 time/month	Self-administered quantitative FFQ. Usual dietary intake during the previous year was assessed at baseline by a self-administered quantitative FFQ of >200 food items. Dietary patterns were determined according to the reported intake of foods of animal origin	Nonvegetarians consumed nonfish meats 1 + time/month and all meats combined (fish included) 1 + time/week	Deaths associated with CHD were identified as ICD-10 I20–25	Mortality data through 31 December 2009, were obtained from the National Death Index. ICD-10 codes for the underlying cause of death were used for causal classification. Unnatural causes of death (ICD-10 letters U, V, W, X, and Y) were considered as censoring events. Deaths associated with IHD were identified as ICD-10 I20–25; CVD deaths, as those starting with the letter I; and cancer deaths, as those starting with the letter C
Tong et al., 2019 (23)	Men and women aged 35 to 59 who were registered with participating general practices, all of whom completed a full questionnaire on their diet, lifestyle, health characteristics, and medical history	Participants who did not eat meat, fish, dairy products, or eggs as self-reported through questions in the form of “Do you eat any meat (including bacon, ham, poultry, game, meat pies, sausages)?”	A full baseline questionnaire	Participants who reported eating meat, regardless of whether they ate fish, dairy, or eggs	CHD (ICD-9: 410–414 or ICD-10: I20–5), including acute myocardial infarction (ICD-9 410, ICD-10 I21); total stroke (ICD-9 430–1, 433–4, 436; ICD-10 I60–1, I63–4); ischaemic stroke (ICD-9 433–4, ICD-10 I63); haemorrhagic stroke (ICD-9 430–1, ICD-10 I60–1)	Participants were followed up via record linkage to records from the UK's health service up to 31 March 2016. Details of events, using the relevant ICD-9 or ICD-10 codes, were obtained from hospital records or death certificates
Key et al., 1999 (22)	Subjects were eligible for analysis if they were aged 16–89 y at recruitment, if they had not been diagnosed with cancer before recruitment (except nonmelanoma skin cancer), and if they provided enough information for classifying diet group and smoking category	People who reported that they did not eat any animal products	Self-reported consumption of meat or fish	The reference group (regular meat eaters) ate meat ≥1 time/week both	Defined by ICD-9 codes: Ischemic heart disease (ICD-9 410–414) & cerebrovascular disease (ICD-9 430–438)	The Adventist Mortality Study: record linkage and personal contact. The Health Food Shoppers Study & the Oxford Vegetarian Study cohort: record linkage with the National Health Service Central Register. The Adventist Health Study; record linkage with the California death certificate file, the USA National Death Index, and church records. The Heidelberg Study; the registrar's office of the last place of residence
Esselstyn et al., 2014 (12)	Self-selection of participants	Whole grains, legumes, lentils, vegetables, and fruit. Subjects were encouraged to take a multivitamin, vitamin B12 supplement & flax seed meal. Caffeine was excluded. Initially all added oils, nuts, processed foods, avocado, excess salt, sugary foods, and drinks were excluded	Self-report adherence to the diet	Not provided	Angina—no definition of CHD—as assessed in radiographic or stress tests	Self-report or, where participant dies, next of kin
Cross-sectional studies						
Fontana et al., 2007 (28)	Vegan subjects were recruited by contacting The St. Louis Vegetarian Society and a Raw Food online magazine. These subjects were consuming a low-calorie low-protein vegan diet, composed of unprocessed and uncooked plant derived foods, for at least 2 years. The second group of subjects comprised endurance runners who were matched with the low-calorie, low-protein, vegan diet group on age, gender, and BMI, and were recruited by contacting local running clubs. The third group of subjects were healthy, sedentary nonobese (BMI 30 kg/m2) subjects, who were eating typical Western diets. These subjects were recruited by local advertising and were matched with the low-calorie, low-protein, vegan diet group on age, gender, and height	Subjects were excluded from the low-calorie, low-protein, vegan diet group if they ate: *1*) animal products including meat, diary, and eggs; and *2*) cooked and processed foods	Subjects were instructed by a research dietitian to record all food and beverage intake, including preparation methods and portion sizes, for 7 consecutive days. Measuring spoon and cup sets, and food diaries with a ruler imprinted on the back cover were provided to the participants to assist with portion size determinations. Supplements were not included in the diet records	*1*) Endurance runners matched on BMI with the low-calorie, low-protein, vegan group; or *2*) nonobese, sedentary subjects consuming typical Western diets	IMT was measured in the anterior wall as the distance from the trailing edge of the adventitia to the leading edge of the intima media; and in the posterior wall as the distance from the leading edge of the intima-media to the trailing edge of the adventitia. The mean of 16 measurements was for the IMT. Examinations and image analyses were performed by a trained sonographer blinded to the subjects’ dietary habits	Carotid artery IMT, which correlates with coronary artery atherosclerosis, was determined by using high-resolution, real-time B-mode ultrasonography with an 11-MHz transducer to image the right and left common carotid arteries. Arteries were scanned in the longitudinal projections over an arterial segment that included 30 mm of the distal common carotid artery. IMT was measured in the anterior wall of the vessel as the distance from the trailing edge of the adventitia to the leading edge of the intima. The average of 16 measurements was taken as the mean IMT. Examinations and image analyses were performed by a trained sonographer who was not aware of subjects’ dietary habits
Randomized controlled trials						
Shah et al., 2018 (25)	Patients from New York University Langone Medical Center with angiographically defined CAD (≥50% lesion in an artery with ≥2‐mm caliber). Exclusion criteria: *1*) history of an eating disorder; *2*) already on vegetarian or vegan diet; *3*) steroids use or nonsteroidal anti‐inflammatory medications other than aspirin; *4*) history of myocardial infarction or coronary artery bypass graft surgery in last 3 months; *5*) presence of infection in last 3 months; and *6*) have a planned staged coronary revascularization or other surgical procedure during study period. Potential participants were also excluded if they had a score of >4 on any of the motivational items or if the relative autonomy index (defined as average of answers for the 6 autonomous items—average of answers for the 6 controlled items) was ≤0 on a treatment self‐regulation questionnaire	Participants in the vegan diet group received 1 point for abstinence from each of the following: *1*) meat/poultry/eggs; *2*) dairy; and *3*) seafood. Participants in the vegan diet group could earn up to 3 points on each of the two 24-hour dietary recalls, and a score of 5 to 6 was defined as adherent to the vegan diet for that week	Participants were instructed to fill out a 4-day food record during the 1 week before the 3 visits to the dietician	American Heart Association–recommended diet with a focus on low fat/fat-free dairy, fish 2+ times a week, and ≤5 oz of animal protein/day. Subjects were provided with groceries, tools to measure dietary intake, and dietary counseling	Secondary outcome: clinical events (e.g., myocardial infarction)	Endothelium activity was measured, as an exploratory end point, in a subset of participants using the EndoPat Device (Itamar Medical Ltd.). Major adverse cardiovascular and cerebrovascular event was defined as the composite of all-cause mortality, myocardial infarction, stroke or transient ischemic attack, and repeat coronary revascularization. Participants were directly asked about interim clinical events during study visits. Source documents were collected, and the reported events were adjudicated by the study investigators blinded to treatment allocation
Wright et al. 2017 (26)	Inclusion criteria: age 35–70 and either obese (BMI 30 kg/m^2^) or overweight (BMI 25 kg/m^2^), with a diagnosis of CHD, hypertension, type 2 diabetes or hypercholesterolemia. Exclusion criteria: diagnoses of life-threatening comorbidities; thyroid disease; coronary artery bypass grafting within 6 weeks; myocardial infarction within 1 month; angioplasty within 6 months; >50% stenosis of the left main coronary artery; unresponsive congestive heart failure; malignant uncontrolled arrhythmias; homozygous hypercholesterolemia; severe mental health disorders; current alcohol or drug misuse; currently smoking; currently pregnant or breastfeeding women, prior bariatric surgery, other conditions that directly affect weight (e.g., lead toxicity, malignancy)	Low-fat, plant-based diet. Included whole grains, legumes, vegetable, and fruits. Participants were advised to eat until satiation. No restriction on calorie intake	3-day recall forms to track dietary indiscretions and exercise	Normal diet: no details reported	Cardiovascular events	Notification on transfer to higher cardiac-related care
						

1 CAD, coronary artery disease; CHD, coronary heart disease; CVD, cardiovascular disease; ICD-9, International Statistical Classification of Diseases, 9th Revision; ICD-10, International Statistical Classification of Diseases, 10th Revision; IHD, ischemic heart disease; IMT, intima media thickness.

### Primary cardiovascular outcomes

Three studies, with at least 73,426 individuals (including at least 7380 vegans) (27), examined the risk of primary cardiovascular events in individuals who followed a vegan diet compared to those who did not (27). Risks of the following events were assessed: total CVD (24), CHD (22–24), acute myocardial infarction (MI) (23), total stroke (22, 23), hemorrhagic stroke, and ischemic stroke (23).

None of the studies reported a significantly increased or decreased risk of any cardiovascular outcome for individuals following a vegan diet. Orlich et al. (24) evaluated 40,907 participants (66.0% women; 13.6% vegan) over a 5–8-year period. The risk of CVD mortality for vegans, compared to non-vegans, was 0.91 (HR; 95% CI, 0.71–1.16), and the risk for CHD mortality was similar (HR, 0.90; 95% CI, 0.60–1.33).

The study cohort of Tong et al. (23), comprising 26,260 participants (75.0% female; 7.0% vegan) who were followed for up to 18.1 years, provided risk estimates for 5 outcomes (acute MI, CHD, stroke, hemorrhagic stroke, and ischemic stroke). Comparing vegans to omnivores, the acute MI risk was 0.77 (HR; 95% CI, 0.46–1.27), the CHD risk was 0.82 (HR; 95% CI, 0.64–1.05), the total stroke risk was 1.35 (HR; 95% CI, 0.95–1.92), the ischemic stroke risk was 1.54 (HR; 95% CI, 0.95–2.48), and the hemorrhagic stroke risk was 1.09 (HR; 95% CI, 0.53–2.26) (23).

Key et al. (22) found no evidence for an increased risk of mortality from CHD or cerebrovascular disease [HR, 0.74 (95% CI, 0.46–1.21) and 0.70 (95% CI, 0.25–1.98), respectively] among 32,519 participants (2.3% vegan) who were followed for an average of 11.7 years.

### Intermediate cardiovascular outcomes

A cross-sectional study (28) of 36 individuals and an RCT (25) of 100 individuals evaluated the association between vegan diets and the intermediate cardiovascular outcomes of carotid artery intima-media thickness (CIMT) and endothelial function, respectively. CIMT was measured using high-resolution real-time B-mode ultrasonography (29) and endothelial function was measured using an EndoPat Device (25).

Common CIMT, a measure of atherosclerotic vascular disease, was reported to be significantly lower in the low-calorie, low-protein, vegan diet group compared to the Western diet group (0.56 ± 0.1 mm versus 0.74 ± 0.1 mm, respectively; *P* < 0.001) (28). In contrast, the EVADE CAD (Effects of a Vegan versus American Heart Association–Recommend Diet in Coronary Artery Disease) trial of participants with a history of angiographically-defined CHD reported no significant change in endothelial function following 8 weeks of adherence to a vegan diet compared to the AHA diet (25).

### Recurrent cardiovascular outcomes

The risk associated with vegan diets was assessed by 2 RCTs (25, 26) and 1 prospective cohort study (12) using recurrent cardiovascularoutcomes, including CHD. These studies had a total of 361 participants, including 57 women and 304 men (aged 57–63 years) (12, 25, 26). No cardiovascular events were observed in the BROAD trial for any participants, regardless of diet (26). In the EVADE CAD trial, no participants in the vegan group had an MI or cerebrovascular event, underwent repeat coronary revascularization, or died during the 8-week study period (25). In the AHA diet group of the same trial, 2 participants had a probable ischemic attack. In the cohort study, a lower risk of worsening cardiac symptoms was observed in individuals who adhered to a vegan diet compared to those who did not (unadjusted RR, 0.16; 95% CI, 0.09–0.29; *P* < 0.001 (12).

### Risk of bias in individual studies and assessment of quality of evidence across studies

According to the AHRQ standards, 3 studies (12, 25, 26) were of poor quality, 4 studies (22–24, 28) were of fair quality, and no studies were of good quality (**Supplemental Tables 3–5**). Shortfalls in study quality in prospective cohort and cross-sectional studies commonly stemmed from low external validity and poor-quality exposure ascertainment methods (i.e., self-reported diet and lack of repeated measurements, leading to increased susceptibility to measurement errors and misclassification) (12, 22–24, 28). In RCTs, study quality was impaired by a high risk of performance bias due to the lack of blinding. Furthermore, only 2 studies performed a power or sample size calculation (25, 26).

The GRADE approach was used to determine the risk of bias across studies where 2 or more studies assessing a particular outcome were identified. Details of the grading based on the current review are indicated in **Supplemental Table 6**. Low to very low evidence was found due to observational studies and downgrading of RCT evidence due to the risk of bias.

## Discussion

To our knowledge, our review provides the most comprehensive assessment of the effect of a vegan diet on incident, recurrent, and intermediate CVD, and adds to the growing body of evidence on the health impacts of vegan dietary patterns (1). There was no significant evidence of an association between adherence to a vegan diet and risks of primary CVD or a CHD event. Although there was some evidence that a vegan diet may prevent recurrent events, the studies were of poor quality as assessed by the Cochrane Collaboration Tool. Findings from 2 studies exploring intermediate outcomes were inconsistent. However, this may be due to the short duration of follow-up (8 weeks) in the EVADE CAD trial, which may have been insufficient to observe a change in endothelial function. The overall evidence for the role of the vegan diet in CVD development or prevention remains weak due to a limited number of high-quality studies.

Another systematic review and meta-analysis indicated that there was low-quality evidence for the association between vegetarian diets and reductions in CHD mortality and incidence, but no evidence for reductions in CVD and stroke mortality (7). This review found inconsistent associations between a vegan diet and different CVD health outcomes. This includes a decreased risk of recurrent CVD events and weak evidence of an increased risk of ischemic stroke with a vegan diet. This inconsistency may reflect a potential protective effect of plant-based foods against CHD and the lower intakes of certain nutrients in vegans versus nonvegans, which may negatively impact cardiovascular health in other ways.

The protective effect may arise because both vegan and vegetarian diets have been associated with significant reductions in established CVD risk factors (7, 29–33), including type 2 diabetes (34), as well as emerging risk factors, such as high-sensitivity C-reactive protein and IL-6 (25, 35). Any favorable effects of the vegan diet on CVD risk factors and cardiovascular functions relative to the omnivore diet are likely to derive from its more optimal macro- and micronutrient contents, including reduced dietary cholesterol and saturated fats (22, 36) and increased plant sterols and fiber (37), as well as its reduced overall energy intake. The vegan diet is also characterized by reduced carnitine and choline consumption, and subsequently lower levels of trimethylamine-N-oxide (TMAO), which is a proatherogenic metabolite produced by intestinal microbiota (38, 39). TMAO, associated with CVD morbidity and mortality, is produced in lower amounts in vegans than in omnivorous individuals (39). However, improvements in CVD risk factors can be made through diets other than veganism, particularly if they emphasize plant food consumption. Importantly, discrepant findings between the 2 studies of intermediate outcomes may have resulted from the EVADE CAD trial using the omnivorous AHA diet as their comparison diet; the AHA diet, which encourages plant foods, is designed to promote cardiovascular health (25).

Conversely, adverse cardiovascular effects associated with vegan diets, such as the suggested increased risk of ischemic stroke, may be partly explained by lower intakes in certain nutrients in vegans compared to nonvegans. These include lower intakes in essential amino acids (40), vitamin B_12_ (41), and long chain n-3 PUFAs (25, 28, 42–44). Additionally, some evidence suggests a protective role of meat-derived factors against stroke (25, 28, 43, 44). Previous studies have reported an increased risk of total stroke (particularly hemorrhagic stroke) among individuals with a low intake of animal products, including animal fat and protein (25, 28, 43, 44), as well as among individuals with low serum cholesterol levels (45, 46). However, food fortification and supplement use may ameliorate these possible adverse effects in vegans.

Our study has several strengths, including a detailed comprehensive search strategy of all available evidence and the assessment of multiple primary, intermediate, and recurrent cardiovascular end points to explore the overall utility of the vegan diet in preventing and managing CVD.

However, it also has some major limitations. First, data insufficiency prevented us from *1*) performing meta-analyses; *2*) assessing publication bias; and *3*) performing subgroup analyses to assess the effects of relevant characteristics (e.g., duration of adherence) on the relationship between the vegan diet and CVD. Low proportions of participants adhered to a vegan diet (a minimum of 2.3% of study participants were vegans) and few participants were included in studies of recurrent outcomes, which limits the power to detect associations and draw conclusions. Second, the definitions of vegan diets and their qualities varied between studies (e.g., some excluded excess salt or added oils). As consumption of unhealthy plant foods like refined grains and starchy vegetables is associated with a greater CHD risk than consumption of healthy plant foods like fruits and nuts, the comparability of diet quality between vegans is an important consideration (47). Third, it should be noted that the studies were conducted in high-income populations, and the studies of recurrent events had varied inclusion criteria, particularly with respect to disease characteristics. This may limit the generalizability of our findings, particularly to low- or middle-income countries, where diet composition and quality may differ and individuals may face unique nutritional challenges and requirements for good health (48). Finally, poor quality diet ascertainment methods using self-report or single dietary measurements may have led to measurement errors, misclassifications, and inaccuracy, and the lack of participant and/or personnel blinding in the included RCTs also increased their susceptibility to performance bias (12, 22–26, 28). Low strength/quality of evidence was also found using the GRADE assessment as a result of observational studies and downgrading of RCT evidence due to the risk of bias.

Whilst society is slowly transitioning to sustainable plant-based diets due to their purported health and environmental benefits, it is important to assess the risks of primary, intermediate, and recurrent cardiovascular outcomes in individuals who follow a vegan diet in order to elucidate its implications on cardiovascular health. Greater knowledge of the cardiovascular impacts of vegan diets may advance global efforts to promote sustainable dietary patterns that are beneficial for both the environment and public health. Future studies should elucidate whether vegan diet quality, demographics, or clinical characteristics modify a potential association between the vegan diet and CVD. Ideally, this should include dose-response analyses of foods, characterizing the various diets and analyses across different CVD outcomes.

In conclusion, to our knowledge, this is the most comprehensive systematic evaluation of vegan diets and cardiovascular health. Transitioning to vegan diets provides significant individual health (e.g., diabetes mellitus) (34) and environmental benefits (1–3). However, our review highlights the need for more studies on the role of vegan diets in cardiovascular health. Further experimental evidence and research in large, ethno-geographically diverse cohorts is warranted to better understand the clinical relevance and public health implications of the vegan diet.

## Supplementary Material

nxab037_Supplemental_File

## Notes

This work was supported by core funding from: the UK Medical Research Council (MR/L003120/1), the British Heart Foundation (RG/13/13/30194; RG/18/13/33946), and the National Institute for Health Research (Cambridge Biomedical Research Centre at the Cambridge University Hospitals NHS Foundation Trust).

Author disclosures: CO-W received funding from Homerton College, University of Cambridge. KRvD received funding by the Gates Cambridge Scholarship (OPP1144) and received funding for publication from the Gates Foundation. All other authors report no conflicts of interest.

The funders had no role in the planning, analysis, write-up, or decision to submit this article.

Supplemental Tables 1–7 are available from the “Supplementary data” link in the online posting of the article and from the same link in the online table of contents at https://academic.oup.com/jn/.

JK and KRvD contributed equally to this work.

Abbreviations used: AHRQ, Agency for Healthcare Research and Quality; CHD, coronary heart disease; CIMT, carotid artery intima-media thickness; CVD, cardiovascular disease; EVADE CAD, Effects of a Vegan versus American Heart Association–Recommend Diet in Coronary Artery Disease; GHG, greenhouse gas; GRADE, Grading of Recommendations Assessment, Development and Evaluation; MI, myocardial infarction; RCT, randomized controlled trial; TMAO, trimethylamine-N-oxide.
