# Supplementary material for: A Systematic Review of the Association Between Vegan Diets and Risk of Cardiovascular Disease
Source: J Nutr. 2021 Apr 8;151(6):1539–52. doi: 10.1093/jn/nxab037 (PMC8169813; doi:10.1093/jn/nxab037)
Supplement: nxab037_Supplemental_File [file nxab037_supplemental_file.docx]

**A systematic review of the association between vegan diet and risk of cardiovascular disease**

**Supplementary Material**

# ***Supplemental Table 1.*** Detailed search strategy with keywords used for PubMed

|  | **Keywords** |
| --- | --- |
| Population | ("humans"[MeSH Terms]) AND |
| Exposure | ("Vegans"[Mesh] OR "Diet, Vegan"[Mesh] OR “vegan*”[All Fields] OR “Plant-based”[All Fields] OR “Plant based”[All Fields]) AND |
| Outcome | (“Acute Coronary Syndrome”[All Fields] OR “Aneurysm”[All Fields] OR “Angina”[All Fields] OR “Aortic Stenosis”[All Fields] OR “Aortic Stenoses”[All Fields] OR “Aortic Valve Insufficienc*”[All Fields] OR “Aortic Valve Stenosis”[All Fields] OR “Aortic Valve Stenoses”[All Fields] OR “Arrhythmia*”[All Fields] OR “Atrial Fibrillation*”[All Fields] OR “Atrial Flutter*”[All Fields] OR “Bradycardia”[All Fields] OR “Cardiac Arrest*”[All Fields] OR “Cardiac Oedema”[All Fields] OR “Cardiac edema”[All Fields] OR “Cardiac Tamponade”[All Fields] OR “Cardiomegal*”[All Fields] OR “Cardiomyopath*”[All Fields] OR “Cardiovascular Disease*”[All Fields] OR “CVD”[All Fields] OR “Cerebrovascular Disease*”[All Fields] OR “Cerebrovascular Disorder*”[All Fields] OR “Cerebral infarction*”[All Fields] OR “Cerebral haemorrhage*”[All Fields] OR “Cerebral hemorrhage*”[All Fields] OR “Commotio Cordis”[All Fields] OR “Coronary Artery Disease*”[All Fields] OR “Coronary Disease*”[All Fields] OR "Coronary Heart Disease"[All Fields] OR “CHD”[All Fields] OR “Coronary Occlusion*”[All Fields] OR “Coronary Restenosis”[All Fields] OR “Coronary Restenoses”[All Fields] OR “Coronary Stenosis”[All Fields] OR “Coronary Stenoses”[All Fields] OR “Coronary Vasospasm”[All Fields] OR “Emboli”[All Fields] OR “Embolism”[All Fields] OR “Endocarditis”[All Fields] OR “Heart Arrest*”[All Fields] OR “Heart Attack*”[All Fields] OR “Heart Block*”[All Fields] OR “Heart Disease*”[All Fields] OR “Heart Failure*”[All Fields] OR “Heart Rupture*”[All Fields] OR “Heart Valve Disease*”[All Fields] OR “Heart Valve Prolapse*”[All Fields] OR “Hypertroph*”[All Fields] OR “Intracranial Haemorrhage*”[All Fields] OR “Intracranial Hemorrhage*”[All Fields] OR “Long QT Syndrome”[All Fields] OR “Mitral Valve Insufficienc*”[All Fields] OR “Myocardial Infarction*”[All Fields] OR “Myocardial Ischemia”[All Fields] OR “Myocardial Ischaemia”[All Fields] OR “Myocardial Reperfusion Injury”[All Fields] OR “Myocardial Stunning”[All Fields] OR “Paroxysmal Dyspnea”[All Fields] OR “Peripheral arterial disease”[All Fields] OR “Pre-Excitation Syndrome”[All Fields] OR “Pulmonary Valve Insufficiency”[All Fields] OR “Pulmonary Valve Stenosis”[All Fields] OR “Pulmonary Valve Stenoses”[All Fields] OR “Pulmonary Heart Disease”[All Fields] OR “Stroke”[All Fields] OR “Sudden Cardiac”[All Fields] OR “Subarachnoid haemorrhage”[All Fields] OR “Subarachnoid hemorrhage”[All Fields] OR “Tachycardia”[All Fields] OR “Thrombosis”[All Fields] OR “Thromboses”[All Fields] OR “Transient Ischaemic Attack”[All Fields] OR “Transient Ischemic Attack”[All Fields] OR “Tricuspid Valve Insufficiency”[All Fields] OR “Tricuspid Valve Stenosis”[All Fields] OR “Tricuspid Valve Stenoses”[All Fields] OR “Ventricular Dysfunction”[All Fields] OR “Ventricular Fibrillation”[All Fields] OR “Ventricular Flutter”[All Fields] OR "Acute Coronary Syndrome"[Mesh] OR "Aneurysm"[Mesh] OR "Angina Pectoris"[Mesh] OR "Aortic Valve Stenosis"[Mesh] OR "Aortic Valve Insufficiency"[Mesh] OR "Arrhythmias, Cardiac"[Mesh] OR "Atrial Fibrillation"[Mesh] OR "Atrial Flutter"[Mesh] OR "Bradycardia"[Mesh] OR "Heart Arrest"[Mesh] OR "Edema, Cardiac"[Mesh] OR "Cardiac Tamponade"[Mesh] OR "Cardiomegaly"[Mesh] OR "Cardiomyopathies"[Mesh] OR "Cardiovascular Diseases"[Mesh] OR "Cerebrovascular Disorders"[Mesh] OR "Commotio Cordis"[Mesh]) OR "Coronary Artery Disease"[Mesh] OR "Coronary Disease"[Mesh] OR "Coronary Occlusion"[Mesh] OR "Coronary Restenosis"[Mesh] OR "Coronary Stenosis"[Mesh] OR "Coronary Vasospasm"[Mesh] OR "Embolism"[Mesh] OR "Endocarditis"[Mesh] OR "Myocardial Infarction"[Mesh] OR "Heart Block"[Mesh] OR "Heart Diseases"[Mesh] OR "Heart Failure"[Mesh] OR "Heart Rupture"[Mesh] OR "Heart Valve Diseases"[Mesh] OR "Heart Valve Prolapse"[Mesh] OR "Hypertrophy"[Mesh] OR "Intracranial Hemorrhages"[Mesh] OR "Long QT Syndrome"[Mesh] OR "Mitral Valve Insufficiency"[Mesh] OR "Myocardial Ischemia"[Mesh] OR "Myocardial Reperfusion Injury"[Mesh] OR "Myocardial Stunning"[Mesh] OR "Dyspnea, Paroxysmal"[Mesh] OR "Peripheral Arterial Disease"[Mesh] OR "Pre-Excitation Syndromes"[Mesh] OR "Pulmonary Valve Insufficiency"[Mesh] OR "Pulmonary Valve Stenosis"[Mesh] OR "Pulmonary Heart Disease"[Mesh] OR "Stroke"[Mesh] OR "Death, Sudden, Cardiac"[Mesh] OR "Subarachnoid Hemorrhage"[Mesh] OR "Tachycardia"[Mesh] OR "Thrombosis"[Mesh] OR "Ischemic Attack, Transient"[Mesh] OR "Tricuspid Valve Insufficiency"[Mesh] OR "Tricuspid Valve Stenosis"[Mesh] OR "Ventricular Dysfunction"[Mesh] OR "Ventricular Fibrillation"[Mesh] OR "Ventricular Flutter"[Mesh] OR "intima-media thickness*"[All Fields] OR "intima media thickness*"[All Fields] OR "FMD"[All Fields] OR "flow mediated dilatation*"[All Fields] OR "flow-mediated dilatation*"[All Fields] OR "endothelial function"[All Fields] OR "endothelial dysfunction"[All Fields] OR "PWV"[All Fields] OR "pulse wave velocit*"[All Fields] OR "pulse-wave velocity*"[All Fields] OR "arter* stiffness"[All Fields] OR "arter* stiffnesses"[All Fields] OR “CAC”[All Fields] OR "coronary arter* calcification"[All Fields] OR “Aort* Stiffness"[All Fields] OR “Aort* Stiffnesses"[All Fields] OR “Pulse Wave Analys*s"[All Fields] OR “Pulse Transit Time*"[All Fields] OR “Pulse Wave Transit Time*"[All Fields] OR “Pulse-Wave Transit Time*"[All Fields] OR "Carotid Intima-Media Thickness"[Mesh] OR "Vascular Stiffness"[Mesh] OR "Pulse Wave Analysis"[Mesh] OR “Intermediate CV*”[All fields] OR “Intermediate cardiovascular”[All Fields]) |

***Supplemental Table 2.*** Overview of included cardiovascular disease (CVD) outcomes

| **Intermediate CVD Outcomes** | **Hard CVD Outcomes** | |
| --- | --- | --- |
| Intima media thickness  Flow mediated dilatation  Endothelial Fysfunction  Pulse-Wave Velocity  Arterial/Artery Stiffness  Coronary Artery Calcification  Vascular Stiffness  Aortic/Aorta Stiffness  Pulse Wave Analysis  Pulse Transit Time  Pulse Wave Transit Time  Intermediate Cardiovascular Disease | Acute Coronary Syndrome  Aneurysm  Angina  Aortic Stenosis  Aortic Valve Insufficiency  Aortic Valve Stenosis  Arrhythmia  Atrial Fibrillation  Atrial Flutter  Bradycardia  Cardiac Arrest  Cardiac Oedema  Cardiomyopathy  Cardiovascular Disease  Cerebrovascular Disease  Cerebral infarction  Cerebral Haemorrhage  Coronary Artery Disease  Coronary Heart Disease  Coronary Occlusion  Coronary Restenosis  Coronary Stenosis  Coronary Vasospasm  Embolism  Heart Arrest | Heart Attack  Heart Block  Heart Failure  Heart Rupture  Heart Valve Disease  Hypertrophy  Intracranial Haemorrhage  Myocardial Infarction  Myocardial Ischaemia  Myocardial Reperfusion Injury  Peripheral Arterial Disease  Pre-Excitation Syndrome  Pulmonary Valve Insufficiency  Pulmonary Valve Stenosis  Pulmonary Heart Disease  Stroke  Sudden Cardiac Death  Subarachnoid Haemorrhage  Tachycardia  Thrombosis  Transient Ischemic Attack  Tricuspid Valve Insufficiency  Tricuspid Valve Stenosis  Ventricular Dysfunction  Ventricular Fibrillation  Ventricular Flutter |

**Supplemental Table 3**. Quality assessment scores of included prospective studies (n = 4) using the Newcastle Ottawa Scale (8)

|  |  | Orlich et al, (1) 2013 | Tong et al, (2) 2019 | Key et al, (3) 1999 | Esselstyn et al, (4) 2014 |
| --- | --- | --- | --- | --- | --- |
| **Selection (max 4 stars)^1^** | *Representativeness of exposed cohort* | *-* | *-* | ★ | **-** |
|  | *Selection of nonexposed cohort* | ★ | ★ | ★ | **-** |
|  | *Ascertainment of exposure* | **-** | **-** | **-** | ★ |
|  | *Outcome absent at study start* | ★ | ★ | **-** | ★ |
| **Comparability (max 2 stars)** | *Comparability of cohorts* | ★★ | ★★ | ★★ | -- |
| **Outcomes (max 3 stars)** | *Outcome assessment* | ★ | ★ | ★ | - |
|  | *Adequate length of follow-up* | ★ | ★ | ★ | ★ |
|  | *Adequacy of follow-up* | ★ | - | ★ | - |
|  | *Total* | 7 | 6 | 7 | 3 |
|  | *AHRQ standard quality* | Fair | Fair | Fair | Poor |

^1^ Each ★ represents a point for a given quality indicator.

AHRQ, Agency for Healthcare Research and Quality; quality categorized as good, fair or poor.

Thresholds for converting the Newcastle-Ottawa scales to AHRQ standards (good, fair, and poor):

**Good quality:** 3 or 4 stars in selection domain AND 1 or 2 stars in comparability domain AND 2 or 3 stars in outcome/exposure domain

**Fair quality:** 2 stars in selection domain AND 1 or 2 stars in comparability domain AND 2 or 3 stars in outcome/exposure domain

**Poor quality:** 0 or 1 star in selection domain OR 0 stars in comparability domain OR 0 or 1 stars in outcome/exposure domain

**Supplemental Table 4.** Quality assessment scores of included cross-sectional study (n = 1) using the New-Castle Ottawa Scale (8)

|  |  | Fontana et al, (5) 2007 |
| --- | --- | --- |
| **Selection (max 5 stars)^1^** | *Representativeness of sample* | ★ |
|  | *Sample size* | **-** |
|  | *Non-respondents* | **-** |
|  | *Ascertainment of exposure (risk factor)* | **-** |
| **Comparability (max 2 stars)** | *Comparability of outcome groups* | ★★ |
| **Outcomes (max 3 stars)** | *Outcome assessment* | ★ |
|  | *Statistical tests* | ★ |
|  | *Total* | 6 |
|  | *AHRQ standard quality* | Fair |

^1^ Each ★ represents a point for a given quality indicator.

AHRQ, Agency for Healthcare Research and Quality; quality categorized as good, fair or poor.

**Supplemental Table 5.** Quality assessment scores of included randomized trials (n = 2) using the Cochrane Collaboration Tool (9)

|  | Shah et al, (6) 2018 | Wright et al, (7) 2017 |
| --- | --- | --- |
| *Random sequence generation (selection bias)^1^* | Low | Low |
| *Allocation concealment (selection bias)* | Low | Low |
| *Blinding of participants and personnel (performance bias)* | High | High |
| *Blinding of outcome assessment (detection bias)* | Low | Unclear |
| *Incomplete outcome data (attrition bias)* | Low | Low |
| *Selective reporting (reporting bias)* | Low | Low |
| *Other sources of bias (other bias)* | Unclear | Unclear |
| *Total risk of bias*  *AHRQ standard quality* | High  Poor | High  Poor |

^1^ Randomized trials were categorized as being at high, low or unclear risk of bias.

AHRQ, Agency for Healthcare Research and Quality; quality categorized as good, fair or poor.

|  | **Effect** | | | | | |
| --- | --- | --- | --- | --- | --- | --- |
|  | *Vegan diets are associated with primary CHD^1^* | | *Vegan diets are associated with primary total stroke^2^* | | *Vegan diets are associated with recurrent CHD^3^* | |
| **GRADE Domain** | **Judgement** | **Justification** | **Judgement** | **Justification** | **Judgement** | **Justification** |
| *Methodological Limitations* | *No serious limitations* | All studies of Fair quality | *No serious limitations* | All studies of Fair quality | Serious limitations | High risk of bias in both studies |
| *Indirectness* | *No serious indirectness* | Both include European/ North American populations, middle-aged participants and majority women | *No serious indirectness* | Both include UK populations | *Serious indirectness* | Differences in the proportion of women, different inclusion/exclusion criteria |
| *Imprecision* | *No serious imprecision* | At least 76,000 individuals included with approximately 4000 events | *No serious imprecision* | Over 50,000 individuals included with 1689 events | *Serious imprecision* | Few patients (100 and 65 participants, respectively) |
| *Inconsistency* | *No serious inconsistency* | Effect estimates are of consistent magnitude | *Serious inconsistency* | Effect estimates in opposite directions. | *No serious inconsistency* | Results were consistent between the studies. |
| *Publication bias* | *Undetected* | The search for studies was comprehensive, limited risk of publication bias | *Undetected* | The search for studies was comprehensive, limited risk of publication bias | *Undetected* | The search for studies was comprehensive, limited risk of publication bias |
| *Overall Quality* | Low(C) | | Very Low (D) | | Very Low (D) | |

**Supplemental Table 6.** Quality of evidence evaluating vegan diets and cardiovascular outcomes

Grade rating was applied when two or more studies assessed the same outcome. GRADE rating – C: Low, the true effect might be markedly different from the estimated effect. Observational studies are initially classified as Low evidence (applicable to risk of primary CHD and total stroke), RCTs are initially classified as High quality (applicablke to risk of recurrent of CHD).

^1^ Based on three studies: Orlich *et al.* 2013, Tong *et al*. 2019 and Key *et al*. 1999 (the latter reporting acute MI risk); ^2^ based on two studies: Tong *et al*. 2019 and Key *et al*. 1999; ^3^ based on two RCTs: Shah *et al*. 2018 and Wright *et al*. 2017.

**References**

1. Orlich MJ, Singh PN, Sabaté J, Jaceldo-Siegl K, Fan J, Knutsen S, Beeson WL, Fraser GE. Vegetarian dietary patterns and mortality in adventist health study 2. JAMA Intern Med 2013;173:1230–8.

2. Tong TYN, Appleby PN, Bradbury KE, Perez-Cornago A, Travis RC, Clarke R, Key TJ. Risks of ischaemic heart disease and stroke in meat eaters, fish eaters, and vegetarians over 18 years of follow-up: Results from the prospective EPIC-Oxford study. BMJ 2019;366:l4897.

3. Key TJ, Fraser GE, Thorogood M, Appleby PN, Beral V, Reeves G, Burr ML, Chang-Claude J, Frentzel-Beyme R, Kuzma JW, et al. Mortality in vegetarians and nonvegetarians: Detailed findings from a collaborative analysis of 5 prospective studies. In: American Journal of Clinical Nutrition 1999;70:516S-524S.

4. Esselstyn CB, Gendy G, Doyle J, Golubic M, Roizen MF. A way to reverse CAD. J Fam Pract 2014;63:356–364b.

5. Fontana L, Meyer TE, Klein S, Holloszy JO. Long-term low-calorie low-protein vegan diet and endurance exercise are associated with low cardiometabolic risk. Rejuvenation Res 2007;10:225–34.

6. Shah B, Newman JD, Woolf K, Ganguzza L, Guo Y, Allen N, Zhong J, Fisher EA, Slater J. Anti-inflammatory effects of a vegan diet versus the american heart association–recommended diet in coronary artery disease trial. J Am Heart Assoc 2018;7:e011367.

7. Wright N, Wilson L, Smith M, Duncan B, McHugh P. The BROAD study: A randomised controlled trial using a whole food plant-based diet in the community for obesity, ischaemic heart disease or diabetes. Nutr Diabetes 2017;7:e256.

8. Modesti PA, Reboldi G, Cappuccio FP, Agyemang C, Remuzzi G, Rapi S, Perruolo E, Parati G. Panethnic Differences in Blood Pressure in Europe: A Systematic Review and Meta-Analysis. PLOS ONE 2016;11:e0147601.

9. Higgins JPT, Savović J, Page MJ, Elbers RG, Sterne JAC. Chapter 8: Assessing risk of bias in a randomized trial. In: Higgins JPT, Thomas J, Chandler J, Cumpston M, Li T, Page MJ, Welch VA (editors). Cochrane Handbook for Systematic Reviews of Interventions version 6.1 (updated September 2020). Cochrane 2020.
